# Recognition of Local DNA Structures by p53 Protein

**DOI:** 10.3390/ijms18020375

**Published:** 2017-02-10

**Authors:** Václav Brázda, Jan Coufal

**Affiliations:** Institute of Biophysics, Academy of Sciences of the Czech Republic v.v.i., Královopolská 135, 612 65 Brno, Czech Republic; jac@ibp.cz

**Keywords:** p53 protein, local DNA structures, protein-DNA interactions

## Abstract

p53 plays critical roles in regulating cell cycle, apoptosis, senescence and metabolism and is commonly mutated in human cancer. These roles are achieved by interaction with other proteins, but particularly by interaction with DNA. As a transcription factor, p53 is well known to bind consensus target sequences in linear B-DNA. Recent findings indicate that p53 binds with higher affinity to target sequences that form cruciform DNA structure. Moreover, p53 binds very tightly to non-B DNA structures and local DNA structures are increasingly recognized to influence the activity of wild-type and mutant p53. Apart from cruciform structures, p53 binds to quadruplex DNA, triplex DNA, DNA loops, bulged DNA and hemicatenane DNA. In this review, we describe local DNA structures and summarize information about interactions of p53 with these structural DNA motifs. These recent data provide important insights into the complexity of the p53 pathway and the functional consequences of wild-type and mutant p53 activation in normal and tumor cells.

## 1. Introduction

p53 is one of the most intensively studied tumor suppressor proteins and its regulation and relation to cancer has been reviewed extensively [1,2,3]. The reason for such interest is obvious; more than 50% of all human tumors contain *Tp53* mutations and inactivation of this gene plays a critical role in malignant transformation [1,3]. As a transcription factor, p53 regulates the expression of many downstream genes in cells undergoing various types of stress and DNA binding is crucial for its function [4,5,6]. It has been demonstrated that p53 binds not only to sequence-specific p53 target sites in linear DNA, but also to local DNA structures such as cruciforms, quadruplexes and triplexes, and to DNA loops, bulged DNA, hemicatenane DNA, etc. In this review, we summarize these data of p53 binding to local DNA structures and how such differential binding influences the activities of p53.

### 1.1. Local DNA Structures

The discovery of B-DNA structure by Watson and Crick in 1953 showed the basic structure of DNA [7]. Further discoveries have led to fascinating findings of the dynamic world of DNA structure, with various DNA forms that differ from this canonical B-DNA structure. These DNA structures, which do not fit the basic double helical model, were originally termed “unusual” DNA structures [8,9,10,11], implying that they are rare structures. However, these local DNA structures are in fact common in the genomes of all organisms and play critical roles in regulating many fundamental biological functions. The negative supercoiling of DNA and protein binding can increase the stability of local conformation and/or induce conformational changes that give rise to various alternative DNA structures, the best described being cruciforms, left-handed DNA (Z-DNA), triplexes and quadruplexes [9,12,13].

#### 1.1.1. Hairpins and Cruciform Structures

**Figure 1:**
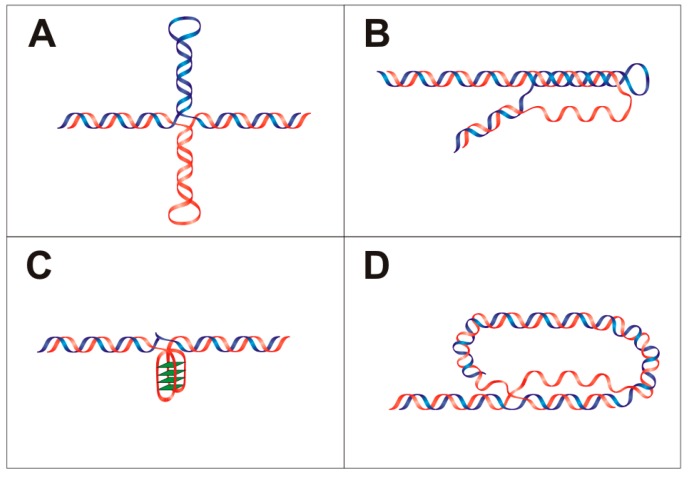
Ribbon scheme of local DNA structures: (**A**) cruciform; (**B**) triplex; (**C**) quadruplex; and (**D**) T-loop. Blue and red represents DNA strands, and G-quartets are highlighted in green rhomboids.

Hairpin and cruciform formation is dependent on DNA sequence and requires inverted repeats in the nucleic acid sequence. The length of this repeat should be six or more nucleotides [14,15]. High non-random occurrences of these inverted repeats were detected in the proximity of breakpoint junctions, promoters and sites of replication initiation [10,16,17] and the probability of cruciform formation can be analyzed by several informatics tools [18,19]. Cruciforms have the ability to affect DNA supercoiling, positioning of nucleosomes in vivo and also the formation and stabilization of other secondary DNA structures [20]. Structurally, they consist of a branch point, a stem and a loop (Figure 1A). Atomic force microscopy has shown two types of cruciform; one has a square planar conformation and the second type consists of a folded conformation, where arms are not perpendicular and neighboring arms are arranged in sharp angle with the B-DNA strand [21,22,23]. Cruciforms are unstable in linear naked DNA because of branch migration [24], although cruciform formation has been identified in both prokaryotes and eukaryotes in vivo [25,26]. A number of proteins with preferential affinity for hairpins and cruciforms have been identified [27]. For example, HMG proteins in various species bind to specific DNA structures [28]; 14-3-3 proteins bind to cruciforms and omission of the 14-3-3 cruciform binding domain reduces initiation of DNA replication [29]. In plants, it was demonstrated that palindromic regions act as hot spots for de novo methylation [30]. The development of new assays that identify DNA cruciforms [31] is expected to increase their reliable detection and further analysis.

#### 1.1.2. Triplexes

A DNA triplex is a non-B-DNA structure consisting of three DNA strands (Figure 1B) where both Watson–Crick and Hoogsteen base pairing are involved [32]. DNA triplexes can be divided into two groups based on the number of DNA strands involved: intramolecular (the third strand is from the same duplex) and intermolecular (the third strand is from a different duplex). In both cases, the third DNA strand binds into the major groove of the DNA duplex. Intramolecular triplexes (also called H-DNA) originate from sequences with homopurine/homopyridine repeats and their role in gene expression regulation has been demonstrated [33]. According to the orientation of the third strand, triplexes are described as parallel (requires N3 protonation for Hoogsteen base pair forming), or antiparallel (does not require acidic conditions). Triplexes in general are relatively unstable compared to DNA duplexes because of a lower number of hydrogen bonds and also due to electrostatic repulsion in negatively charged phosphate backbones. Triplex stability can be enhanced by the presence of Mg^2+^ ions, which relieves the electrostatic repulsion [34]. Triplex formation is affected by the length of triplex-forming sequence, type of triplet, ionic conditions, molecular crowding and chromatin accessibility [35]. Triplex-forming sequences are abundant, especially in eukaryotes [36,37]. Most polypurine-polypyrimidine DNA tracks are found in introns, promoters, and 5′ and 3′ untranslated regions. It was shown that triplex-forming sequences are enriched in genes related to cell communication and signaling [38]. In human cells, H-DNA structures were immunodetected by antibodies with triplex specificity [39]. Results have shown the importance of triplexes in different cellular processes; for example H-DNA structure creates an effective block for *Taq* DNA polymerase [40], an H-DNA forming sequence stops replication fork progression on plasmid in *S. cerevisiae* and triplex formation reveals single stranded DNA (Figure 1D), potentially leading to DNA breaks [41]. It was shown that GAA/TTC triplet expansion in the first intron of the frataxin gene forms triplex structure [42] and this triplet expansion is associated with Friedreich’s ataxia [43,44]. DNA triplet repeats can adopt several unusual DNA structures, including hairpins, triplexes, or quadruplexes [45]. Moreover, purine repeats capable of forming stable DNA triplex structures are associated with neurological disorders [46].

#### 1.1.3. Quadruplexes

Another known non-B DNA structure is the quadruplex (Figure 1C) [47,48]. The tetrameric arrangement of guanines was demonstrated by crystallographic methods as the G-quartet in 1962 [49]. One of the first quadruplexes to be characterized in detail was from human telomere sequences [50]. Nowadays, there are numerous software tools for quadruplex prediction [51,52,53]. The existence of quadruplex forming sequences correlates with functional genomic domains and over 40% of human genes have G-rich areas [51]. Both DNA and RNA can form quadruplex structures and the presence of a G-quadruplex was shown in the 5′ untranslated region of the majority of mRNAs [54]. G-quadruplexes have been detected in vivo with specific antibodies and by quadruplex specific interacting fluorescent compounds [55,56] that bind and stabilize G-quadruplex structures in DNA and RNA [48,57]. It has also been demonstrated that quadruplexes are important for gene expression [58,59].

#### 1.1.4. T-Loops

Mammalian telomeres form large duplex loops (T-loops, Figure 1D) in vivo [60]. T-loop formation requires the presence of TRF2 protein, a telomere junction consisting of a 3′ single strand overhang and TTAGGG repeats bordering the double strand part of the telomere [61]. Similarly, evolutionarily divergent organisms protect the ends of their DNA sequences via looping, for example *Oxytricha nova* [62] and *Trypanosoma brucei* [63]. Telomere looping is probably a common mechanism for protecting the ends of linear chromosomes.

## 2. Interaction of p53 with DNA

**Figure 2:**
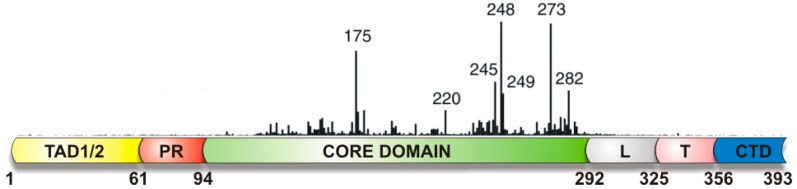
Schematic structure of p53. p53 is composed from several domains: a transactivation domain (TAD) with TAD1 and TAD2 subdomains, a proline-rich region (PR), the core domain (CD, DNA sequence specific binding domain), a flexible linker region (L), a tetramerization domain (T), and the regulatory domain at the extreme carboxyl terminus (CTD). The vertical bars indicate the relative missense-mutation frequency in human cancer for each residue (Tp53 Mutation Database [69]. Adapted from [66].

p53 controls an extensive transcriptional network, providing response to cellular and environmental stresses or damage [64,65]. p53 is a multifunctional protein, however its main function as a tumor suppressor is provided by its interaction with DNA. p53 protein structure has been extensively reviewed [66,67,68]. The p53 protein contains a natively unfolded amino-terminal transactivation domain, which can be further subdivided into two subdomains, followed by a proline-rich region. The structured DNA-binding and tetramerization domains are connected through a flexible linker region. The C-terminal part of the protein is the regulatory domain (Figure 2). It is generally accepted that p53 contains two DNA binding domains: (a) the core domain; and (b) the basic C-terminal domain.

### 2.1. Sequence-Specific Interaction

p53 regulates target gene expression by either activation or inhibition of p53-responsive promoters. Critical for its function is the DNA sequence-specific binding [70]. The p53 target sequence comprises two copies of a 5′-RRRC(A/T)(T/A)GYYY-3′ sequence, which can be separated by a spacer of 0 to 13 bp [71]. p53 interacts with its target sequence as a tetramer [72,73]. Interestingly, natural p53 target sequences are highly heterogeneous [74] and different bases may be located anywhere except at positions 4 and 7 in each half site [75,76]. The core domain of the p53 protein is mainly responsible for DNA target sequence interactions [77,78], but C-terminal modification is mandatory for effective binding of the full-length protein to the target sequence [79]. The length of the DNA spacer between decamers is another important determinant for p53 binding and transactivation, and the insertion of nucleotides between the two decamers leads to lower transactivation. p53 transactivation is also possible in yeast or human cell systems from just one decamer [80]. Even though the core domain of p53 is able to bind target sequences on its own, the full-length protein is required for efficient specific DNA binding and for the effective searching for the p53 target site in long DNA [81,82,83]. It was shown that p53 searches for the target by combining three-dimensional diffusion and one-dimensional sliding along the DNA [6,84]. The C-terminal domain mediates fast sliding of p53, while the core domain samples DNA by frequent dissociation and reassociation, allowing for rapid scanning of long DNA regions [85]. Using single-molecule fluorescence microscopy, it was demonstrated that p53 protein sliding was significantly accelerated by the presence of divalent cations and depended on the sequence of DNA, suggesting that p53 possesses two sliding modes with different diffusion coefficients [84,86]. This proposal is supported by molecular simulation, showing that p53 diffuses along nonspecific DNA via rotation-uncoupled sliding with its C-terminal domain, whereas the core domain repeats cycles of dissociation and association [87]. Target sequences and their recognition by p53 has been extensively studied and reviewed [76,88,89,90,91,92,93], therefore we will focus in this review on p53 binding to DNA with non-B DNA structures.

#### 2.1.1. DNA Bending by p53 Protein Binding

**Figure 3:**
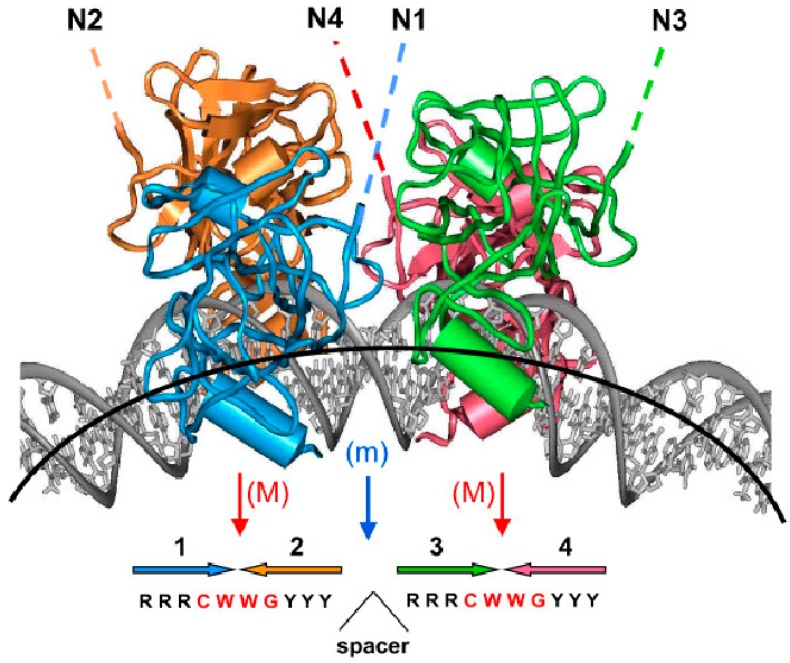
A structure with four p53 DBD domains bound to bent DNA (adapted from [95], *Proc. Natl. Acad. Sci. USA*, **1999**, *96*, 1875–1880). Four p53 core domains bound to bent DNA. The figure is based on the computational model that was further corroborated by gel electrophoresis experiments [100]; the overall DNA bend is ~40°. The red arrows show the major-groove bending (M) in the CWWG tetramers; the blue arrow denotes the minor-groove bend (m) in the center of the site. The lateral positioning of p53 DBDs on the external side of the DNA loop and the degree of DNA bending imply that, in principle, the p53 tetramer can bind to nucleosomal DNA. The dashed lines indicate that the N-termini of the p53 tetramer (N1–N4) are accessible for interactions with trans-activation and trans-repression factors. Large colored arrows (1–4) at the bottom of the figure indicate the orientations of the four p53 target site subunits.

It was demonstrated that p53 interaction with DNA target sites causes their bending, and that bending angles correlate with binding affinity to these response elements [94]. In vitro assays have shown that the p53 core domain bends linear B-DNA up to 52 degrees for an ideal p53 target sequence and that the bending of symmetrical p53 sequences is about 50 degrees for the CDKN1A/p21 promoter; 37 degrees for the RGC site; and 25 degrees for the SV40 promoter binding site [94]. Angles are higher for full-length p53 protein, meaning that amino acids flanking the core DNA binding domain take part in DNA binding/bending [95]. It was also demonstrated that binding cooperativity is determined by the structural properties of this region, particularly the torsional flexibility of the CWWG motif; transactivation properties of p53 target sequences are therefore strictly determined by their intrinsic physical properties [96]. Moreover, it was demonstrated that the conformational switch influences DNA binding off-rates independently of affinity [97,98]. Molecular dynamic simulations have identified sequence-dependent differential quaternary binding modes of the p53 tetramer interfacing with DNA and showed direct interactions of the p53 C-terminal region with DNA [99]. A structure with four p53 core domains bound to bent DNA is shown (Figure 3. These results point to the importance of DNA structure for effective p53 binding to its target.

Palecek et al. showed preferential binding of wild-type p53 protein to supercoiled DNA [101]. It was later demonstrated that hot-spot mutant p53 proteins (R175H, G245S, R248W, R249S, R273C, R273H and R282W) also have the ability of wild-type p53 to preferentially bind supercoiled DNA, while the same DNA molecules in linear or relaxed circular DNA were poorly bound [102]. Using chromatin immunoprecipitation, prior binding of mutant p53 to target sites in superhelical DNA was detected also in cells [103]. Interestingly, supercoiled DNA can stabilize different non-B DNA structures including cruciforms, triplexes and quadruplexes [9].

#### 2.1.2. p53 Binding to p53 Target Site Enhanced by Cruciform Extrusion

**Figure 4:**
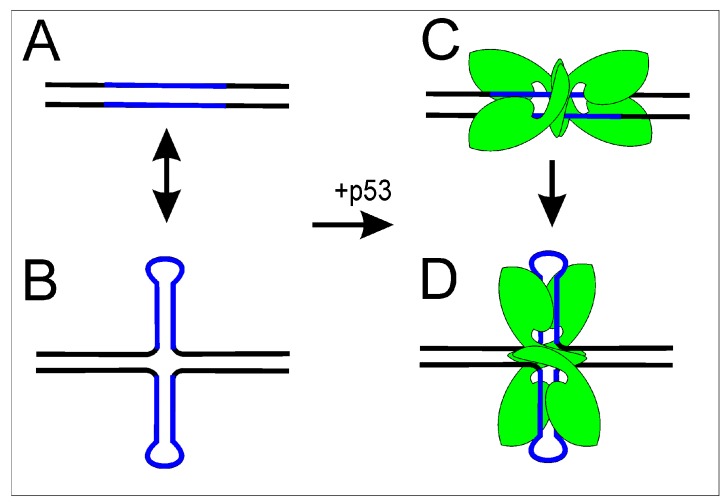
Scheme of p53 binding to its response sequence in linear and superhelical states. p53 target (blue) can be presented in: (**A**) linear DNA; or (**B**) cruciform DNA. p53 (green) binds to its target in: linear (**C**); or cruciform (**D**) structure with preference to cruciform structure probably due to better accessibility and/or stability of the complex.

There is a strong correlation between the inverted repeat in the p53 target site and enhancement of p53 binding to DNA [104,105]. p53 binds p53 response sites capable of forming cruciform structures in topologically constrained DNA with a remarkably higher affinity compared to asymmetric p53 response sites [106]. These results implicate DNA topology as having an important role in modulation of the p53 regulon. p53 binding to supercoiled DNA with and without a p53 target sequence has been demonstrated [101]. Moreover, the cruciform structures in p53 target sequences are preferred by p53 both in vitro [107] and *in vivo* as demonstrated by chromatin immunoprecipitation [108] and by transactivation assay with full-length p53 protein [109]. It seems that both better accessibility of the p53 target sequence and higher stability of the complex play roles in the preferential binding of p53 to target sequences in a cruciform structure (Figure 4).

### 2.2. p53 Binding to Local DNA Structures

It was demonstrated that p53 interacts with a set of non-canonical DNA structures: p53 preferentially binds to duplexes with mismatches, cruciforms [110], bent DNA [81], structurally flexible chromatin [111], hemi-catenated DNA [112], DNA bulges, three way or four way junctions [113], telomere T-loops [114] and superhelical DNA [106,115,116].

#### 2.2.1. Bulged DNA and Mismatches

When two similar DNA molecules containing some non-homologies undergo recombination, some single based mismatches and extra base bulges may be left behind. Their presence must be signaled for repair. These mistakes in base pairing and bulges may also arise as a consequence of errors in replication or repair of DNA damage. p53 and its C-terminal domain can form a complex with base bulges, insertion/deletion mismatches, and extra bases on one strand. Complexes formed in this way are quite stable [117]. Comparison of bulge, regular and mismatch sequences (every mismatch possible) using filter binding analysis identified the most attractive binding substrate for p53 as 3-cytosine bulge and then mismatches C/C, A/G, regular, A/C and G/T. When comparisons were made on agarose gels, the results were slightly different; 3-cytosine bulge was still the most attractive, followed by C/C, A/G, A/C and T/T, both experiments were performed at low ionic strength (50 mM KCl or NaCl) [110]. At higher ionic strength, the interaction with double stranded linear DNA was quite weak and insertion of a G/T mismatch had almost no effect on p53 binding, but insertion of an A/G mismatch enhanced binding almost threefold compared to linear DNA [118].

#### 2.2.2. Holliday Junctions and Cruciforms

**Figure 5:**
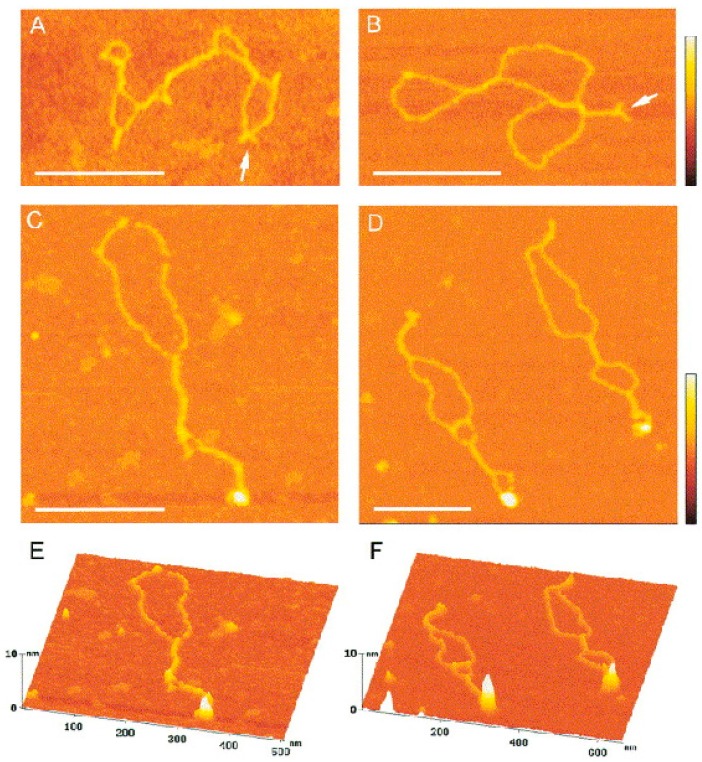
Binding of p53CD to supercoiled DNA forming cruciform structures. (**A**,**B**) Scanning force microscopy images of the sc pXG(AT)34 plasmid DNA bearing an (AT)34 sequence; small arms forming Y-shapes (arrows) are clearly visible. (**C**–**F**) SFM images of complexes formed between p53CD and sc pXG(AT)34 plasmid DNA at a molar ratio of 2.5; the bound proteins are clearly seen as large protuberances on surface plots (**E**,**F**). The scale bars represent 200 nm. The color bars represent 5 nm (reprinted from [123], with permissions from *Elsevier*).

Lee et al. demonstrated p53 interaction with DNA Holliday junctions. Using electron microscopy, it was shown that DNA junctions are bound mostly by p53 trimers or tetramers (46%) and less by dimers (15%) or monomers (6%), but are also bound by higher oligomeric p53 forms (33%) [119]. *T4 endonuclease* and *T7 endonuclease I* are well characterized junction resolvases [120]. They interact specifically with the junction and cleave these four way junctions by introducing nicks at asymmetrically related positions across the junction. Preincubation of four way junctions with p53 before adding one or other endonuclease leads to more effective cleavage of the junction; for *T4 endonuclease VII* about nine-fold lower concentration of enzyme was required for the same cleavage efficiency [119]. Although the molecular mechanism for this phenomenon is unclear, these results indicate the importance of p53 in the recognition of DNA junctions. Negative superhelicity and protein interactions with DNA lead to stabilization of non-B DNA structures. It was demonstrated that p53 binds with higher affinity to both negatively and positively supercoiled DNA [101,121]. Interestingly, cruciform structures, which can be stabilized by DNA supercoiling, share two or three structural parts (four-way junction and the stem) with Holliday junctions. It is therefore not surprising that p53 interacts with topologically constrained DNA [105,122] and with cruciforms [108,123]. By electron and scanning force microscopy it was demonstrated that the p53 core domain binds to cruciform in plasmids with an inserted sequence (AT)34 (Figure 5) [123,124]. The strong correlation between negative superhelix density changes and p53 binding enhancement to cruciform-forming DNA sequences pointed to the importance of three-dimensional DNA structure for effective p53 binding [105]. Interestingly, in silico analysis of the *CDKN1A* gene promoter with three p53 binding sites situated at positions ~1400, ~2300 and ~4500 [125] shows a correlation between inverted repeat presence and effective p53 binding demonstrated by chromatin immunoprecipitation [108].

#### 2.2.3. Hemicatenane DNA

Hemicatenanes are thought to be important intermediates for DNA replication, repair and recombination. This structure is created by a denaturated DNA duplex that renatures in the presence of HMGB1 or HMGB2 [126] and is resolved by topoisomerases [127]. Hemicatenane DNA is made by DNA reassociation of fragments containing a poly(CA)poly(TG) repeat [126]. It was shown that p53 shares similar preferences for structures such as extra base bulges, UV irradiated DNA, DNA modified with the anticancer drug cisplatin, three stranded DNA structures, Holliday junctions and DNA minicircles with HMGB1 protein [81,117,128]. p53 protein isolated from baculovirus infected insect cells could bind hemicatenate DNA forming three bands (three types of complexes) on agarose gels. Complex I is formed by p53 binding to a high affinity site (DNA loop and hemicatenane) and complex II contains p53 bound to low affinity sites (linear segments outside of the DNA loop) within complex I. When complex I has both low affinity sites occupied by p53, then it forms complex III [112]. Interestingly, p53 with deletion of the last 30 amino acids can interact with hemicatenane DNA [112].

#### 2.2.4. Telomeric T-Loop and Single Strand Overhangs

Mammalian telomeres are organized into large duplex loops in vivo (T-loops) [60]. In vitro, these structures are created in the presence of TRF2 at a telomeric junction consisting of a 3′ single stranded overhang of at least one TTAGGG repeat neighboring the double stranded part of the telomere [61]. It is also possible that a portion of the C-rich strand of the double and single stranded telomeric junction may invade the duplex, creating a Holliday junction-like structure at the base of the T-loop [61]. When T-loops created by TRF2 are incubated with p53, 88% have p53 bound exclusively at the T-loop junction and it seems that p53 interacts with this structure as a tetramer or as two tetramers, but when the TTAGGG sequence is present in double stranded linear form, there is no/very low binding—depending on protein concentration. TTAGGG presented as double stranded DNA in a plasmid is also not very attractive for p53, whereas p53 binds single stranded TTAGGG with high affinity [114]. Another interesting finding is that p53 also has strand transfer activity [129] that may assist TRF2-mediated T-loop formation or p53 may even create these T-loops. In vitro experiments showed that p53 does not mediate T-loop formation itself, but T-loop formation is increased when TRF2 and p53 cooperate (compared to TRF2 T-loop formation). TRF2 transformed about 13% of DNA molecules into T-loops, but together with p53 this increased to 24% and both proteins were often detected together (86%) at the T-loop junction [114].

#### 2.2.5. Triplexes

Triplex-forming sequences occur in many gene promoters, for example IL2R, DNA-POL1 and MYC [130], which suggests a potential role of triplexes in transcription. A structure-selective DNA binding of wild-type and G245S mutant p53 proteins on the intermolecular triplex (dT)50.(dA)50.(dT)50 has been described [131]. Binding of wild-type p53 on plasmid DNA containing triplex-forming sequence demonstrated that p53 prefers a superhelical form of plasmid with triplex extrusion. The use of antibodies to the p53 N- and C-terminal domains showed that the C-terminus of p53 probably plays a key role in triplex TAT binding [131]. Recently, comparative analyses of p53 binding to triplex DNA in vitro and in vivo have been published [132]. The influence of triplex structure on p53 transactivation in cells was measured by luciferase reporter assays in H1299 cells with pCDNAp53 vector and transactivation of the candidate p53 target genes with TAT triplex-forming motifs in promotor region was validated by RT-PCR [132]. Comparative analyses show that p53 binding to triplex DNA is comparable to recognition of hairpin structure. Comparison of gel-shifts and ELISA with full-length protein and isolated parts of the protein—core domain and C-terminal domain—showed surprisingly that all constructs are able to bind a TAT triplex, however the affinity of the full-length and C-terminal domain of p53 is significantly higher than the affinity of the p53 core domain, showing the crucial importance of the C-terminal domain in triplex recognition [132].

#### 2.2.6. Quadruplexes

Recent research has highlighted the importance of quadruplexes in multiple cellular processes, including DNA replication, telomere maintenance and the binding and activity of transcription factors [133,134]. Compared to wild-type p53, mutant p53 proteins do not bind or bind only weakly to p53 target sequences [135,136]. However, both mutant p53 (G245S, R248W and R273H) and wild type p53 have the ability to interact with quadruplex structures in vitro [137]. It was shown that mutant p53 proteins modulate transcription on a global scale through their binding to intronic and intergenic sequences predisposed to form non-B DNA structures [138]. There was also enrichment of mutant p53 bound to regions from 1 kb upstream to 1 kb downstream of transcription start sites [137]. This frequent association of mutant p53 with transcription start site regions corresponds with a significant overlap with CpG islands (about 90% overlap). Mutant p53 was found to bind regions of DNA containing 20–21 bp long G-rich motifs, which are predisposed to formation of local DNA structures. About 75% of mutant p53 binding regions comprise G-quadruplex motifs and mutant p53 can also stabilize quadruplex folding in vitro as revealed by circular dichroism spectroscopy [137]. Association of p53 with G/C-rich DNA motifs could be mediated by other transcription factors (SP1 and ETS1) that interact with these motifs [139,140]. Reciprocal coimunoprecipitation discovered only low levels of these proteins forming a complex with p53, and direct evaluation of mutant p53, ETS1 and SP1 binding regions showed that only a small fraction of mutant p53 binding regions serve also as a binding site for EST1 or SP1, or for both of these transcription factors [137]. It has been shown recently by chromatin immunoprecipitation that p53 binds the G-quadruplex forming sequence in the *Myc* promoter and by luciferase transactivation assay in cell lines that this p53 binding leads to represssion of transcription [141]. It was also demonstrated that p53 binds to telomeric G-quadruplexes and that binding is stronger with an increased number of telomeric repeats. Furthermore, p53 strongly favors G-quadruplexes folded in the presence of potassium ions over those formed in sodium ions, thus indicating the telomeric G-quadruplex conformational selectivity of p53. N-methyl mesoporphyrin IX (a quadruplex-stabilizing ligand), increases quadruplex recognition by p53. Experiments with separated p53 domains and with selective oxidation of the p53 core domain show that both p53 DNA binding domains are important for its G-quadruplex recognition [103].

## 3. Conclusions

**Figure 6:**
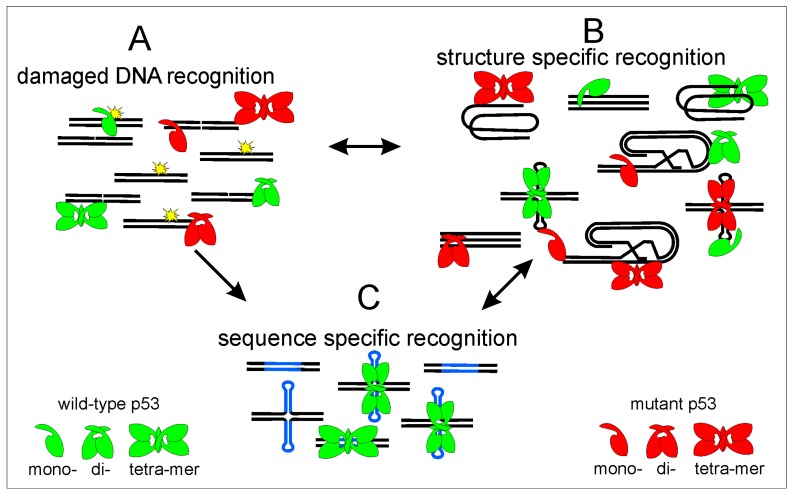
Scheme of p53 DNA recognition to different targets: p53 recognizes damaged DNA (**A**); different DNA structures (**B**); and sequence-specific p53 target sequences (**C**). Wild-type protein is shown in green, mutant p53 by red color, DNA strands by black, p53 target sequence by blue, DNA damage is marked by DNA breaks and by yellow. p53 protein has affinity to different DNA targets, after DNA damage p53 binding to DNA leads to p53 stabilization and protection before degradation, these processes could lead to both increased association with local DNA structures (**B**) and increased p53 binding to p53 targets in linear and cruciform structure (**C**). However, p53 binds to p53 target sequences effectively only as a tetramer and p53 core domain mutants bind to p53 target sequences only weakly or not at all. Therefore, we suppose that the equilibrium among different p53 DNA binding properties is moved to preferential binding to different local DNA structures especially to DNA triplexes and quadruplexes where the C-terminal part of p53 plays the critical role. However, even wild-type p53 protein binds to local DNA structures, so probably exact equilibrium during basic cell processes is essential for correct p53 function.

DNA binding is fundamental for the ability of p53 to act as a tumor suppressor. p53 has diverse activities in transcription, repair, recombination, replication and chromatin accessibility. These functions are realized through versatile modes of p53 interaction with DNA (Figure 6). p53–DNA interaction with p53 target sites is highly sensitive to DNA topology and architectural features are a key parameter contributing to p53 DNA affinity and specificity [105,108,135]. It was also demonstrated in vitro and in vivo that p53 has a strong preference for conformationally flexible CTG·CAG trinucleotide repeats [142], cruciform structures, triplexes, quadruplexes, DNA damage, etc. Many studies have shown that p53 binds to target sequences as a tetramer (Figure 6C) [143,144]. The known “gain of function” role of p53 mutants can therefore be partly caused by heterodimerization of the mutant and wild-type proteins in a dysfunctional complex [145,146]. However, another possibility for “gain of function” roles of p53 mutants can be due to introduction of an imbalance among p53 sequence-specific and structure-specific binding. The three-dimensional organization of DNA is a critically important and basic feature of organisms and local structure of DNA is often a target for different proteins [27,147,148]. The recognition of non-B DNA structures plays important roles in gene regulation and is critical for DNA replication. It seems that p53 acts as a DNA topology-modulating factor [142] and this role could be another basic part of the importance of this protein in the prevention of cancer development.

An important part of the complex role of p53 in cancer development is DNA binding of mutant p53. Despite the partial or complete loss of sequence specific DNA binding, mutant p53 proteins can induce or repress transcription of mutant p53-specific target genes and various mutant p53 proteins can bind to oligonucleotides mimicking non-B DNA structures [149]. Mutant p53 proteins bind selectively and with high affinity to local DNA structures, this binding depends on the spatial arrangement of the DNA, but not on DNA sequence. It has been proposed that DNA structure-selective binding of mutant p53 proteins is important for mutant p53 interaction with nuclear matrix attachment region DNA elements (that are important for large-scale chromatin structural organization) and also for transcriptional activities mediated by mutant p53 [135,150,151]. According to recent data indicating a crucial role of the C-terminal domain of p53 for binding to triplex DNA [132] and a similarly important role of the C-terminal domain in binding to G-quadruplex [141], as well as the results of hot-spot p53 mutants binding selectively to G-quadruplex [103], the importance of the C-terminal domain in p53 binding to local DNA structures is an emerging concept. The importance of the intrinsically disordered C-terminal domain in the complex process of p53 regulation has been recently reviewed [152]. Interestingly, not only the C-terminal domain but also the core domain of p53 is important for mutant p53/DNA interactions. The biological implications of mutant p53 binding to DNA have been reviewed [153] and both wild-type and mutant p53 are able to bind quadruplex DNA [103,137].

It was demonstrated that the stability of p53 is enhanced by its binding to local DNA structures. These structures can therefore remarkably change p53 dependent transactivation as was demonstrated for triplex structures in promotor regions [132]. Besides its effect on gene transcription, local DNA structures recognition by p53 can play important role in DNA repair, replication and recombination. For example, it was shown that p53 binding to subtelomeric regions leads to prevention of accumulation of DNA damage [154] and it was also shown by genome-wide analyses that p53 is associated with many different genome locations, including sites not associated with transcriptional control [155]. p53–DNA interactions are highly versatile as demonstrated by the wide spectrum of p53 sequence- and structure-specific targets. Flexibility of p53 is probably one of its most important features, allowing subtle cellular regulation which lead to its key role in basic biological processes and protection against cancer development. An interplay of p53 DNA recognition among different DNA targets seems to be a crucial aspect of p53 function. A deeper understanding of p53’s DNA sequence-specific and structure-specific binding properties in the context of topology and chromatin will help to elucidate the exact role of this “guardian of the genome”.
