# Correction: Brázda, V. and Coufal, J*.* Recognition of Local DNA Structures by p53 Protein. *Int. J. Mol. Sci.* 2017, *18*, 375

**DOI:** 10.3390/ijms19092737

**Published:** 2018-09-13

**Authors:** Václav Brázda, Jan Coufal

**Affiliations:** The Czech Academy of Sciences, Institute of Biophysics, Královopolská 135, 612 65 Brno, Czech Republic; jac@ibp.cz

The authors wish to make the following corrections to their paper [1]: We have recently been made aware of some errors and omissions in the following paragraph of our recent paper.

Section 2.2.5, lines 2–6 currently read as follows:

A structure-selective DNA binding of wild-type and G245S mutant p53 proteins on the intermolecular triplex (dT)50.(dA)50.(dT)50 has been described [131]. Binding of wild-type p53 on plasmid DNA containing triplex-forming sequence demonstrated that p53 prefers a superhelical form of plasmid with triplex extrusion. The use of antibodies to the p53 N- and C-terminal domains showed that the C-terminus of p53 probably plays a key role in triplex TAT binding [131].

To set the scientific record straight, we would like to replace this with the following:

Structure-selective DNA binding of wild-type p53 protein on the intermolecular triplex (dT)50.(dA)50.(dT)50 has been described [131]. Binding of wild-type p53 on plasmid DNA containing triplex-forming sequence demonstrated that p53 prefers a superhelical form of plasmid with triplex extrusion. The use of antibodies to the p53 N- and C-terminal domains showed that the C-terminus of p53 probably plays a key role in triplex TAT binding [131].

The original Reference 131 has been deleted, and all the reference numbers after it have been changed accordingly. References 132–155 are now numbered 131–154.

The authors would like to apologize for any inconvenience caused to the readers by these changes. The changes do not affect the scientific results. The manuscript will be updated and the original will remain online on the article webpage, with a reference to this Correction.
